# Prolactin and Dehydroepiandrosterone Levels in Women with Systemic Lupus Erythematosus: The Role of the Extrapituitary Prolactin Promoter Polymorphism at −1149G/T

**DOI:** 10.1155/2015/435658

**Published:** 2015-10-25

**Authors:** Edward L. Treadwell, Kenneth Wiley, Beverly Word, William Melchior, William H. Tolleson, Neera Gopee, George Hammons, Beverly D. Lyn-Cook

**Affiliations:** ^1^Brody School of Medicine, East Carolina University, Greenville, NC 27834, USA; ^2^National Human Genome Research Institute, Rockville, MD 20892, USA; ^3^FDA-National Center for Toxicological Research, Jefferson, AR 72079, USA

## Abstract

Systemic lupus erythematosus (SLE) has shown an association with high levels of prolactin, low levels of dehydroepiandrosterone (DHEA), and induction of inflammatory cytokines in the serum of patients with the disease. This preliminary study examined the relevance of a −1149G/T functional single-nucleotide polymorphism (SNP) (rs1341239) in the promoter of the extrapituitary prolactin gene in a cohort of African American and European American women with lupus. Examination of this SNP revealed that the −1149TT genotype was correlated with higher levels of prolactin in serum and prolactin gene expression (*p* = 0.0001) in peripheral blood mononuclear cells (PBMCs). Lower levels of DHEA in serum were demonstrated in lupus patients (*p* = 0.001); those with the −1149TT genotype had the lowest levels of DHEA. Furthermore, a small subset of women who were on DHEA therapy and had a TT genotype showed a significant decrease in prolactin gene expression and lower disease activity scores (SLEDAI). Lupus patients, particularly African Americans, had significantly higher levels of IL-6 (*p* = 0.0001) and TNF-*α* (*p* = 0.042). This study suggests that the −1149TT genotype may be a risk factor for lupus and may predict who could possibly benefit from DHEA therapy; therefore, these results should be validated in a larger cohort with all ethnic groups.

## 1. Background

Systemic lupus erythematous (SLE) is a complex debilitating and fatal autoimmune disease affecting between 1.4 and 2.0 million Americans [1]. Hormonal, infectious, and environmental factors have been implicated in the etiology of the disease [2, 3]. Although the precise etiology of SLE remains elusive, the pathogenesis is often attributed to the development of antinuclear or anti-double stranded- (ds-) DNA autoantibodies [2, 4, 5]. Of the three types of lupus (systemic, discoid, and drug-induced), it is estimated that 70% of diagnosed lupus cases are systemic in which a major organ is affected in 50% of the cases, whereas discoid lupus accounts for approximately 10% of all other cases [1]. Genetic susceptibility to lupus has also been demonstrated by the fact that 20% of people with lupus will have a close relative (parent or sibling) who already has lupus or may develop lupus and, furthermore, approximately 5% of the children born to individuals with lupus will develop the illness [1]. Two noteworthy and perplexing aspects of SLE are its biases towards women and non-Europeans. SLE exhibits a female to male bias during prepubescence that increases from 4.5 : 1 in adolescence to 8–12 : 1 in adults and then declines to 2 : 1 in adults >60 years of age [2]. Women of childbearing age account for approximately 80–90% of SLE patients [6]. African American women experience 2–10-fold increased risk for developing SLE compared to European American women, develop symptoms at an earlier age, and have more severe symptoms and increased mortality [1, 2, 7, 8]. In fact, as many as 1 in every 250 African American women is diagnosed with lupus [9].

Although few biomarkers have been discovered in early stages of lupus development, a number of biological changes have been noted in animal models or molecular epidemiological human studies [10]. High levels of prolactin have been associated with SLE and these levels have been correlated with a single-nucleotide polymorphism in the promoter of the prolactin gene [11]. However, a number of studies have shown conflicting results with this observation, likely due to different ethnic groups used in the various studies [12, 13].

Prolactin participates in a number of important functions in the body: it performs as a hormone, mainly due to its pituitary production, and it acts as a cytokine. It is prolactin's role as a cytokine, in which it participates in autocrine and paracrine actions that suggests an important role in the immune system. Prolactin is also secreted by immune cells and its receptor belongs to the family of cytokine receptors type 1 [14]. Although prolactin expression by the pituitary and other tissues utilizes the same gene, the promoters, regulatory regions, transcriptional control mechanisms, and final mRNA transcripts are tissue-dependent [15]. Studies have suggested its role in immunomodulation; however, the actual role of prolactin in the immune system remains unclear.

In addition to high levels of prolactin, lupus patients have low serum levels of dehydroepiandrosterone (DHEA). DHEA and its metabolite, dehydroepiandrosterone sulphate (DHEAS), are the major androgens secreted by the adrenal glands and are the precursor for estrogen and testosterone [16]. DHEA exerts antiproliferative and anti-inflammatory effects, and it modulates immune function [17]. In addition to low levels of DHEA in lupus patients, studies have shown low levels of DHEA in other inflammatory diseases [18].

One of the overall objectives of this study was to determine if the polymorphism in the promoter of the extrapituitary prolactin gene modulates expression levels of prolactin, particularly in African American women whose inclusion has been limited in other studies, and whether this polymorphism plays a role in patients' response to DHEA therapy. Levels of DHEA, DHEAS, estrogen, and testosterone in women with and without lupus were examined. Finally, this study also investigated whether ethnic differences were noted in IL-6 or TNF-*α* levels between European American women and African American women.

## 2. Methods

### 2.1. Human Blood Samples

Blood samples were obtained and processed as previously published from our laboratory [19] from a cohort of patients, after obtaining informed consent, who had been diagnosed with lupus according to the American College of Rheumatology criteria and were currently on routine therapy in addition to treatment with or without DHEA. This study consisted of a total of 256 patients, 87 African American females with lupus, 76 healthy age-matched controls (will be designated as nonlupus); 25 European American females with lupus, 33 healthy age-matched controls (will be designated as nonlupus); 10 African American males with lupus, 13 healthy age-matched controls (will be designated as nonlupus); and 5 European American males with lupus, 7 age-matched healthy controls (will be designated as nonlupus). These patients were a part of a larger LUPUS study at the Brody School of Medicine-East Carolina University, Greenville, NC. For consistency, all blood samples were obtained between 9:00 am and 12:00 pm, with a previous period of fasting and resting. Whole blood was collected in two portions: 10 mL whole blood was collected in plain or serum separator tubes for serum hormone and cytokine analyses and an additional 10 mL whole blood was collected in heparinized tubes for molecular biological studies. Blood and serum samples were shipped after collection to the National Center for Toxicological Research (NCTR) for molecular and clinical chemistry analyses. This study received IRB approval from East Carolina Brody School of Medicine and the FDA Research Involving Human Subject Committee (RIHSC).

### 2.2. Prolactin T→G^−1149^ Polymorphism

Genotyping was conducted using restriction fragment length polymorphism (RFLP) analyses and confirmed by DNA sequencing. Genomic DNA was isolated from peripheral blood mononuclear cells (PBMCs) using a modified QIAmp DNA Blood Maxi kit (QIAGEN, Valencia, CA). The polymorphism at position −1149 of the promoter of the extrapituitary PRL gene was amplified with the following primers: F-GAAGTTGAGCCTCAGGATGG and R-CTCAACAGCTTCTCAGTCA-ACA. PCR-RFLP analysis with* ApoI *digestion was conducted on the sequence for prolactin (GENBANK Accession number AF068856:GATAACCTGG AGAAAGGAGG AAAGATAATT TTATGGAGTT AGAGAGACA) that contained the prolactin polymorphism in the promoter region. All polymorphisms were confirmed with automatic sequencing.

### 2.3. RNA Isolation and Quantitative Real Time PCR

RNA was extracted as described previously [18] by using a PAXgene RNA kit (QIAGEN, Valencia, CA). After extraction, all RNA samples were tested for their integrity and concentration using a Bio-Rad Experion Automated Electrophoresis System (BIO-RAD, Hercules, CA). cDNAs were synthesized from total RNA extractions using a Clontech Advantage RT-for-PCR Kit (Clontech, Mountain View, CA). Prolactin expression analysis was conducted using a Bio-Rad IQ5 quantitative Real Time Polymerase Chain Reaction Detection System (BIO-RAD, Hercules, CA). GAPDH was used as an endogenous control. qRT-PCR conditions were as follows: 50°C for 2 minutes and 95°C for 10 minutes (95°C for 15 seconds, 62°C for 30 seconds, and 72°C for 30 seconds) for 47 cycles. Relative quantitation of prolactin and TNF-*α* mRNA expression was normalized to GADPH and fold changes were calculated using the 2^−ΔΔCT^ method. Primers utilized for prolactin, TNF-*α*, and GAPDH are listed below:

 Prolactin R: 5′ CGG CGC GGT CAA ACA GGT CT 3′.
 Prolactin F: 5′ ACC AGG AAA AGG GAA ACG AAT GCC 3.
 GAPDH-F: 5′ CCACCCATGGCAAATTCCATG 3′.
 GAPDH-R: 5′ TCTAGACGGCAGGTCAGGTCC 3′TNF-alpha.
 TNF-*α*-F: 5′-CTT CTC CTT CCT GAT CGT GG-3′.
 TNF-*α*-R: 5′-GCT GGT TAT CTC TCA GCT CCA-3′.

### 2.4. Circulating Hormones and Cytokines

Testosterone, estradiol, prolactin, and DHEA-sulfate were assayed using Siemens RIA “Coat-A-Count” methods (Los Angeles, CA) and DHEA was assayed with Diagnostics Systems Laboratories (Webster, TX) using a coated tube RIA method. All tubes were counted on a PerkinElmer Cobra 5005 gamma counter (Shelton, CT). TNF alpha and IFN gamma were assayed using Antigenix America (Huntington Sta., NY) ELISA kits. All plates were read on the BioTex Instruments (Winooski, VT) Elx808 plate reader using Gen5 software for curve analysis and calculations.

### 2.5. Statistics

Differences in the frequencies of the GG, GT, and TT genotypes were analyzed using Fisher's exact test. For this study, a comparison analysis was conducted for each of the specific genes, serum levels, and cytokine profiles. The comparisons included patients with SLE compared to the control population and African Americans with SLE compared to European Americans with SLE. A two-tailed Mann-Whitney *t*-test was used to determine if significant differences existed. To compare values, *p* values of <0.05 were considered significant. All analyses were performed using GraphPad Prism (version 4) software (La Jolla, CA).

## 3. Results

**Figure 1:**
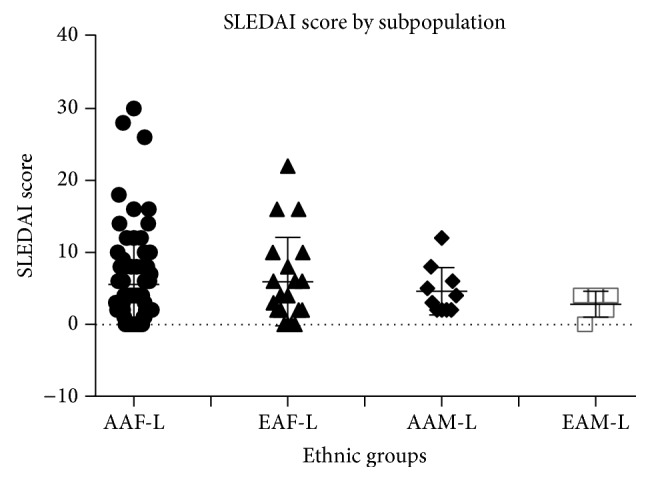
SLE disease activity index (SLEDAI) by population. African American (AAF-L) and European American (EAF-L) women with lupus have the highest SLEDAI scores compared to African American and European American men with lupus.

Figure 1 shows the individual differences in the SLEDAI score by subpopulations in this study.

**Figure 2:**
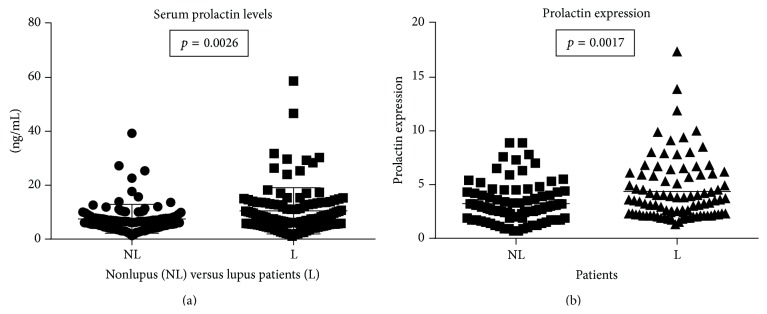
Prolactin expression and serum levels. Patients with lupus have significantly higher serum levels of prolactin (*p* = 0.0026) and expression at the mRNA level in PBMCs (*p* = 0.0017), although individual differences are shown among the patients.

The highest disease activity within our study was found in African American and European American women with lupus. This study had a higher number of African American women compared to other published studies investigating the −1149G/T polymorphism. Higher levels of prolactin were noted in the serum of lupus patients (*p* = 0.0026) (Figure 2(a)). Prolactin gene expression levels were also higher in PBMCs from lupus patients (*p* = 0.0017) compared to nonlupus controls (Figure 2(b)).

**Figure 3:**
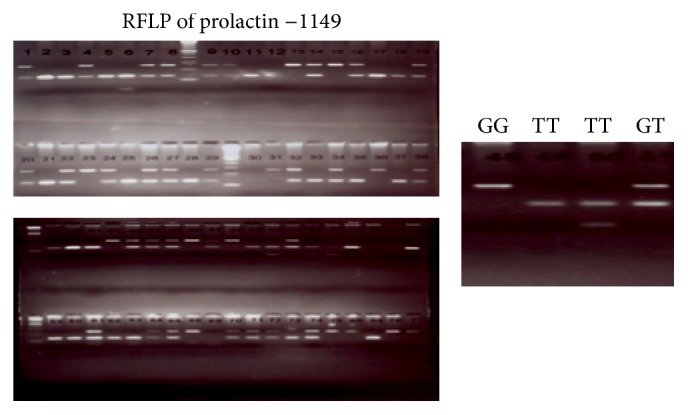
Restriction fragment length polymorphism (RFLP) of prolactin −1149. RFLP analysis with* ApoI* digestion yields three fragment lengths to distinguish the three genotypes, GG, GT, and TT.

Figure 3 shows a representative electrophoretic gel depicting RFLP analysis of the prolactin −1149 SNP.

**Figure 4:**
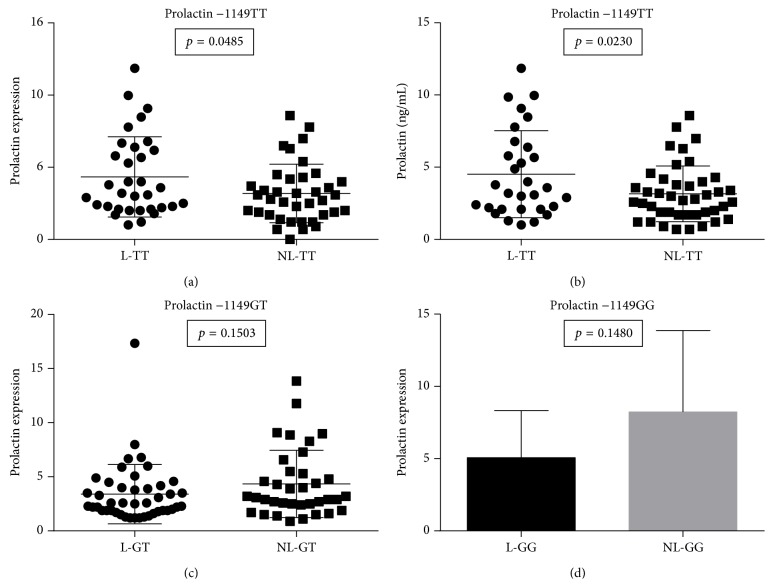
The genotype TT prolactin levels and expression. A significant number of lupus patients with the TT genotype demonstrated higher levels of prolactin expression (a) (*p* = 0.0485) and protein serum levels (b) (*p* = 0.0230). There were no significant differences in expression or serum levels in the other genotypes GT (c) and GG (d).

Figure 4(a) shows that the genotype −1149TT was correlated with higher prolactin gene expression (*p* = 0.0485) in lupus (L) patients compared to age-matched nonlupus (NL) patients. An increase in prolactin levels was also noted in the serum prolactin protein (*p* = 0.0230) of these patients (Figure 4(b)). However, the −1149GT (*p* = 0.1503) and −1149GG (*p* = 0.1480) genotypes did not significantly correlate to any differences in prolactin gene expression levels in PBMCs from lupus patients when compared to age-matched nonlupus patients (Figures 4(c) and 4(d)).

**Figure 5:**
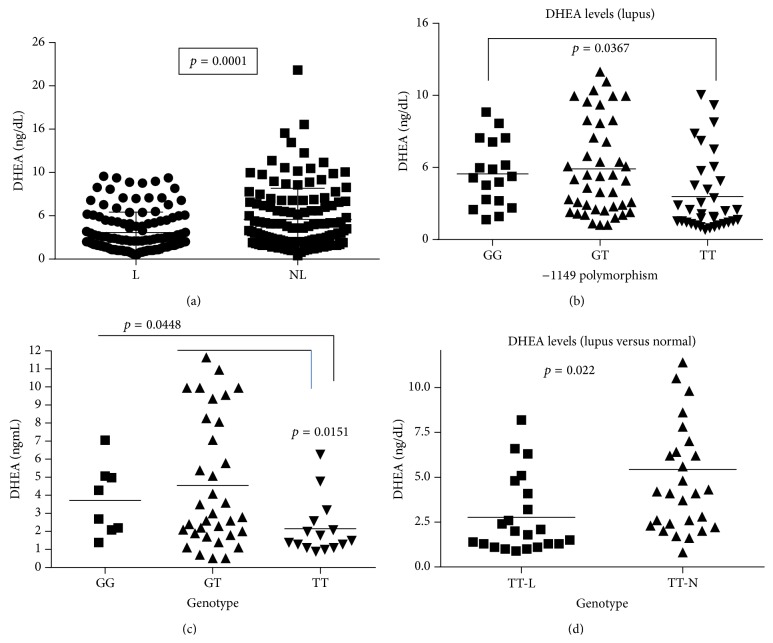
Serum levels of DHEA. (a) shows that lupus patients had a significant lower level of serum DHEA (*p* = 0.0001). (b) shows that the TT genotype had the lowest level of DHEA compared to GT or GG in lupus patients. Furthermore, (c) demonstrates that African American women with lupus and the TT genotype had the lowest level of DHEA. (d) shows that the serum DHEA level in TT genotype in African American women was significantly lower when compared to their normal age-matched nonlupus patients (*p* = 0.022).

In addition to high levels of prolactin, low levels of DHEA were detected in the serum of lupus patients. Patients with lupus had lower levels of DHEA when compared to age-matched controls (*p* = 0.0001) (Figure 5(a)). Lupus patients with the TT genotype had lower serum levels of DHEA than patients with the GG genotype (*p* = 0.0367) (Figure 5(b)). Furthermore, African American women with lupus with the TT genotype had significantly lower DHEA levels (*p* = 0.0151), when compared to female African American lupus patients with the GG or GT genotype (Figure 5(c)) or when compared to age-matched controls (*p* = 0.022) (Figure 5(d)).

**Figure 6:**
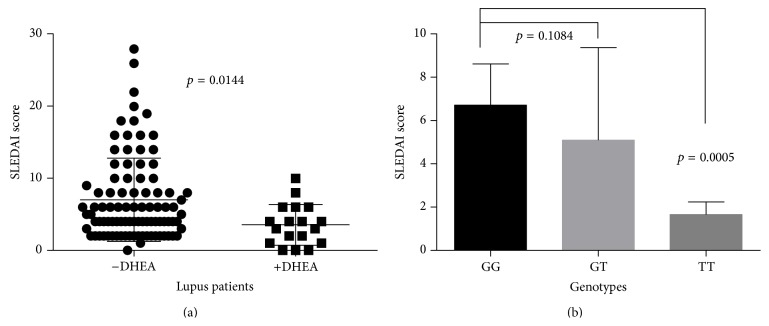
SLEDAI score and DHEA therapy. (a) shows that lupus patients on DHEA therapy had lower SLEDAI scores than patients not on therapy. (b) demonstrate that those lupus patients with a TT genotype and on DHEA therapy had lower disease activity scores compared to those with GT and GG genotypes.

When DHEA was given as therapy, patients had a lower disease activity index (*p* = 0.0144) (Figure 6(a)) and those with the TT genotype had an even lower disease activity index (*p* = 0.0005) compared to the GG genotype (Figure 6(b)). In addition, DHEA lowered the levels of prolactin in a selected group of patients (data not shown).

**Figure 7:**
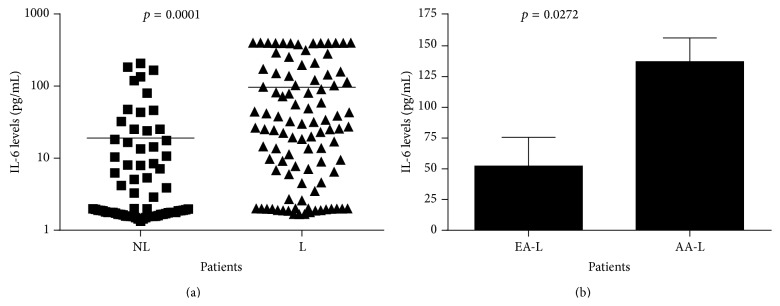
IL-6 serum levels in lupus and nonlupus patients. (a) shows that lupus patients have a significantly higher level of IL-6 compared to age-matched controls (*p* = 0.0001); however, (b) African American women with lupus have an increased level of IL-6 compared to European American women with lupus (*p* = 0.0272).

DHEA is thought to regulate proinflammatory cytokines; therefore, low DHEA levels could play a role in expression of high levels of cytokines, such as IL-6 and TNF-*α*. Our data showed (Figure 7(a)) higher levels of IL-6 expression in lupus patients (*p* = 0.0001) and that African American women with lupus had higher levels than European American women (*p* = 0.0272) (Figure 7(b)).

**Figure 8:**
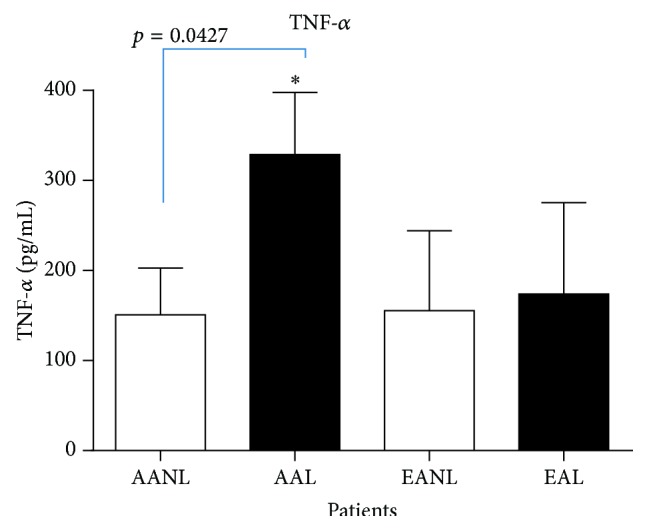
Tumor necrosis factor-alpha (TNF-*α*) serum levels. African American women with lupus (AAL) had the highest level of TNF-*α* compared to European American women with lupus (*p* = 0.0427).

African American women with lupus also had a higher level of TNF-*α* (*p* = 0.0427) (Figure 8).

**Figure 9:**
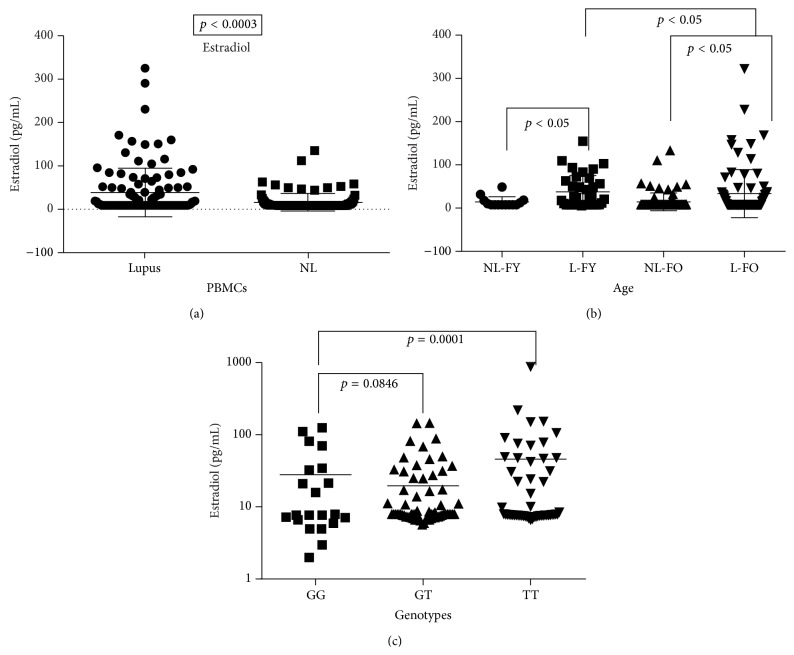
17*β*-estradiol levels in serum. Estradiol levels in serum were significantly higher in lupus patients (a) (*p* = 0.0003). (b) However, women with lupus older (FO) than 50 had higher levels compared to women less than 50 (FY). (c) Lupus patients with the TT genotype had higher levels than women with the GG genotype.

In addition to high levels of prolactin and low levels of DHEA, results from this study revealed a significant increase in serum estradiol in lupus patients (Figure 9(a)) (*p* = 0.0003). There was a significant difference in estradiol levels in premenopausal (female young (FY)) (<50 years of age) and postmenopausal women (female old (FO)) (>50 years of age) (*p* < 0.05) when compared to their nonlupus counterpart at each menopausal status (*p* < 0.05), respectively (Figure 9(b)).  Figure 9(c) further showed that lupus women with the TT genotype have a significant higher level of estradiol (*p* = 0.0001) when compared to the GG genotype. There was no difference in estradiol levels in lupus males compared to nonlupus males (data not shown).

**Figure 10:**
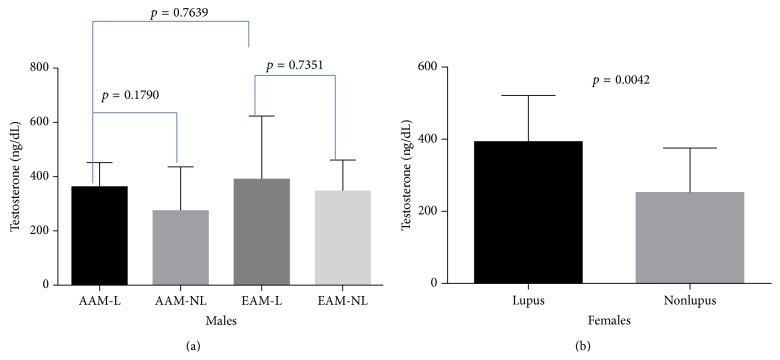
Testosterone levels in serum. (a) No difference was found in testosterone between the small numbers of males with lupus in this study; however, we did notice a higher level of testosterone in a small number of women less than fifty years of age.

No significant differences were found in testosterone levels between African American males and European American males with lupus (*p* = 0.7639) (Figure 10(a)); however, a small number of young women with lupus demonstrated higher levels of testosterone (Figure 10(b)).

## 4. Discussion

Prolactin's role in human autoimmune diseases remains a largely unexplored area in which research is greatly needed, particularly when investigating whether its involvement in different ethnic groups may cause different biological outcomes based on an individual's exposure and lifestyle factors. Prolactin's exact role in the physiology and pathogenesis of autoimmune diseases, such as lupus, has not been totally clarified. This protein acts as both a hormone and a cytokine depending on its biological context [14]. Prolactin is considered a cytokine due to its secretion by immune cells and by the fact that its receptors belong to a family of cytokine receptors type I.

Although prolactin is mainly expressed by the pituitary gland, extrapituitary promoter expression has been shown in a number of other organs [20]. Both pituitary and extrapituitary prolactin share the same gene but are under different promoters. Extrapituitary expression of prolactin is cell-specific and is independent of the Pit-1 transcription factor, which induces pituitary expression of prolactin [21]. The extrapituitary promoter has been shown to contain a functional single-nucleotide polymorphism (SNP) at −1149G/T (rs1341239) in the GATA sequence. Studies have shown that the G allele leads to higher prolactin levels in lymphocytes in serum [22] and the GG genotype was associated with systemic lupus [23]; however, our study showed a different finding. The TT genotype was associated with higher expression of prolactin in lupus patients which may be due to the majority demographic in this study being African American women. Previous studies were done in different ethnic groups or had a very limited number of African American women while our study population was predominantly African American women.

This study further demonstrated that the lupus cohort in this study had a higher serum level of prolactin in their PBMCs in comparison to age-matched nonlupus patients. Not only was the TT genotype associated with higher expression of prolactin, but also these patients showed decreased levels of DHEA. DHEA and its metabolite dehydroepiandrosterone sulfate (DHEAS) are the most abundant circulating human adrenal steroids [16]. The critical role of low levels of DHEA in autoimmune diseases, such as lupus, rheumatoid arthritis, and other inflammatory diseases, remains an underexplored area of research; however, studies have indicated its involvement in improving overall immune function [24]. Animal studies have shown that high levels of proinflammatory cytokines, such as IL-6 and TNF-*α*, are restored to normal levels by DHEA administration [25]. It is believed that DHEA decreases TNF*α* and IL-6 through inhibition of NF-*κ*B activation [26]. Higher levels of serum IL-6 were found in lupus patients in this study, and African American women had the highest level when compared to European American women and males. African American women also had the highest level of TNF-*α* when compared to nonlupus and lupus European American women and men in general. This study showed that, in a subgroup of women on DHEA therapy, those lupus patients with the TT genotype had lower levels of IL-6 expression compared to those patients on DHEA therapy with GG or GT genotypes. Furthermore, women with a TT genotype on DHEA therapy had a lower disease activity score. These results need further investigation with larger populations; however, they could explain the conflicting results shown in a number of studies or clinical trials using prasterone (DHEA) [27–29].

This investigation has confirmed earlier reports of low levels of DHEA in lupus patients and high levels of prolactin. This study is the first to demonstrate a correlation between low levels of DHEA and high levels of prolactin and the TT genotype, particularly in African American women. A number of other studies that examined the extrapituitary polymorphism at −1149 showed that the GT was correlated with high levels of prolactin; however, those studies were done in populations other than African American women. Another finding in this study was that high levels of estradiol were also associated with the TT genotype and lupus patients in general had a higher level of estrogen when compared to their nonlupus counterparts, regardless of their menopausal status.

These findings in African Americans may give valuable insights into the lack of positive outcomes from various treatments and increased morality rates at younger ages. IL-6 and TNF-*α* expression levels were significantly higher in African American women. High levels of IL-6 have been associated with kidney damage in humans and in a number of animal models [30]. SLE is a chronic inflammatory disease that may affect any organ system in the body but it is the leading cause of kidney disease associated with death in young women [31]. IL-6 has both pro- and anti-inflammatory properties and increased IL-6 expression is a common response to tissue injury and organ failure [32]. Although this pathway has been targeted for novel therapeutic approaches, most drugs fail due to toxicity or other severe side effects.

These results warrant larger studies with diverse populations to fully understand the role of the functional single-nucleotide polymorphism −1149G/T in the promoter of the extrapituitary prolactin gene and its effects on autoimmunity. Also this study demonstrates that further studies should be conducted on the mechanisms by which DHEA therapy is decreasing disease activity indexes in selected lupus patient with the TT genotype. Furthermore, more research is needed to determine if genotypes of prolactin extrapituitary promoter can be translated into clinical benefit, using a personalized medicine approach, for patients identification for DHEA therapy.
